# Thinking Outside the Nation: Cognitive Flexibility’s Role in National Identity Inclusiveness as a Marker of Majority Group Acculturation

**DOI:** 10.3390/bs15040498

**Published:** 2025-04-09

**Authors:** Anna Medvetskaya, Andrew G. Ryder, Marina M. Doucerain

**Affiliations:** 1 Département de Psychologie, Université du Québec à Montréal, Montréal, QC H3C 3P8, Canada; medvetskaya.anna@courrier.uqam.ca; 2 Psychology Department, Concordia University, Montréal, QC H3G 1M8, Canada; andrew.ryder@concordia.ca; 3 Lady Davis Institute, Jewish General Hospital, Montréal, QC H3T 1E1, Canada

**Keywords:** national identity, identity inclusiveness, majority group acculturation, cognitive flexibility, latent class analysis, mainstream cultural group

## Abstract

In superdiverse societies like Canada, characterized by high levels of cultural and ethnic plurality, national identity boundaries are often blurry. While policies may officially promote inclusiveness, public discourse on national identity is frequently dominated by mainstream groups, whose willingness to expand these boundaries plays a crucial role in fostering minority inclusion. Despite the importance of inclusivity for social cohesion, little is known about what enables majority group members to adopt a more inclusive national identity. This study addresses this gap by exploring the role of cognitive flexibility in facilitating an acculturative shift toward inclusiveness. Using latent class regression analysis (*N* = 202), we identified two distinct national identity profiles: one more inclusive and the other more exclusive. We also examined how factors such as ethnic vs. civic views on national identity, acculturation orientations toward integration, and personal identification with traditional English Canadian vs. multicultural identity representation shape these profiles. Our findings revealed that higher cognitive flexibility was positively associated with the likelihood of belonging to the more inclusive profile. This study contributes to a limited body of work on majority group acculturation, offering insights into how cognitive flexibility may encourage a broader and more inclusive national identity. Implications for policy and social cohesion are discussed.

## 1. Introduction

National identity inclusiveness refers to the degree to which people recognize and incorporate diverse cultural, ethnic, and social groups in their mental representation of the nation (Pehrson et al., 2009). In other words, national identity inclusiveness characterizes the expansiveness of potential answers to questions such as “who is a real Canadian?” In the context of superdiversity, where societies are characterized by a high degree of cultural, ethnic, and linguistic plurality (Vertovec, 2007), national identity boundaries become increasingly blurry. Despite official policies that often promote inclusiveness, the public discourse around national identity often remains dominated by the mainstream group—the historically dominant ethnocultural population. Migrants and minority ethnocultural group members may strive to integrate into the mainstream society and adopt the national identity, but their inclusion into that national identity representation is also contingent upon the willingness of mainstream group members to share that identity (Grigoryan & Ponizovskiy, 2018; Indelicato & Martín, 2023). Thus, inclusion is as much about minority group efforts as it is about the mainstream group attitudes toward inclusion or exclusion of others (Kunst et al., 2021). Put differently, national identity inclusiveness is a direct reflection of majority group acculturation, i.e., “the cultural and psychological changes that current or former majority-group members experience and the cultural styles they adopt as a result of contact with people self-identifying as immigrants or ethnic minority-group members living in the same society” (Kunst et al., 2021, p. 486). The ingroup projection model suggests that mainstream members typically define national identity as including those who resemble them. However, overcoming this bias and becoming more inclusive fosters social cohesion (Bonikowski & DiMaggio, 2016; Citrin et al., 2012; Doucerain et al., 2018). The key question is then: what enables mainstream cultural group members to make this acculturative shift toward inclusiveness? To address this question, we first examine the relationship between national and mainstream identities, followed by an exploration of national identity inclusiveness and majority group acculturation. We then introduce cognitive flexibility as a psychological mechanism that may facilitate inclusive national identity representations. Finally, we present our analysis and results, followed by a discussion of the findings, their implications, limitations, and directions for future research.

## 2. National and Mainstream Identities

Research on national identity is abundant in political, social, and psychological fields. As a result, our understanding of national identity can differ considerably depending on the research angle. Here, we refer to Anderson’s (1983, 2020) oft-cited definition as a feeling of belonging to an *imagined political community*. While the feeling of belonging is quite real, a political community, argues Anderson, is imagined because it is based on shared feelings of identification with the nation and connectedness with fellow members despite not knowing them personally. Moreover, this feeling of connectedness implies horizontal relationships with these others, where the ideal of equality fosters a deep sense of attachment among millions of people despite actual inequalities. This definition underscores the significance of psychological perceptions of nationality, where people feel a psychological bond with other ingroup members and perceive a shared destiny.

A common distinction in the literature differentiates between *civic* and *ethnic* national identity. A civic national identity is defined by shared values, democratic participation, and political membership, whereas an ethnic national identity is based on shared ancestry, language, and cultural heritage (Yogeeswaran & Dasgupta, 2015). In societies where the ethnic interpretation of identity is dominant, national identity often overlaps with the cultural identity of the mainstream group. In such cases, national identity is defined in ways that emphasize the historical, linguistic, and cultural elements most pertinent to the mainstream group (Devos et al., 2010; Smith, 1991). As Anderson (1983, 2020) argues, the mainstream group plays a crucial role in constructing a shared sense of national belonging through symbols, myths, and collective memories that align with their experiences and perspectives. This dominant narrative often marginalizes minority groups, framing them as outsiders or secondary members of the national community (Wimmer, 2002). Consequently, the mainstream group’s control over the national identity discourse can lead to exclusionary practices and reinforce social hierarchies (Joppke, 2005).

## 3. National Identity Inclusiveness

Beyond civic and ethnic distinctions, national identity can also vary in inclusiveness—that is, the extent to which individuals recognize and incorporate diverse cultural groups into their representation of the nation. While an ethnic national identity is often exclusive—since it defines belonging based on heritage—exclusivity can also manifest within a civic framework if certain groups are still perceived as outsiders due to cultural differences (e.g., Pehrson & Green, 2010). Conversely, an inclusive national identity can exist within both ethnic and civic frameworks, as it reflects a willingness to recognize diversity within national belonging. This conceptualization differs from broader superordinate identities, such as identification with all of humanity or *global identity* (McFarland et al., 2012), which transcend national boundaries. While inclusive national identity shares some overlap with these constructs—particularly in emphasizing humanitarian values—it remains rooted within the national context rather than extending to a universal human ingroup. Unlike global identity, which transcends national boundaries, an inclusive national identity emphasizes national belonging while incorporating diverse cultural perspectives.

National identity inclusiveness is crucially important in superdiverse settings where dominant symbols, myths, and collective memories will routinely not speak to the experience of a large proportion of the population. Conceptualizing national identity through the lens of the mainstream group thus often undermines the inclusive potential of a nation’s identity, exacerbating tensions in multicultural societies. As a case in point, Canada has enshrined multiculturalism in its constitution, mandating respect and equal civic participation of all cultural groups. Nevertheless, minority groups are expected to adopt a dominant Canadian identity that reflects the mainstream group (Brosseau & Dewing, 2018). Thus, their success in doing so does not depend solely on their willingness, but also on the willingness of mainstream group members, to include others in representations of the national identity and when answering, “who is a real Canadian?” (Kunst et al., 2021; Pinto et al., 2020).

## 4. Majority Group Acculturation

Such willingness to be included and to include reflects the adaptive dynamics of acculturation—the process of mutual adjustment of majority and minority groups upon prolonged contact (Berry, 1997, 2005). Majority acculturation refers to how members of the mainstream cultural group in a society adapt their attitudes, behaviors, and values due to sustained interactions with minority group members living in the same society (Kunst et al., 2021), which can lead to a more inclusive or exclusive view of national identity. Majority group acculturation^1^ unfolds along two dimensions: inclusion of non-mainstream groups within national identity and maintenance of the mainstream culture (Kunst et al., 2021; Lefringhausen et al., 2021; Lefringhausen & Marshall, 2016). However, contrary to globalization-based acculturation frameworks (e.g., multicultural acquisition vs. ethnic protectionism), mainstream culture maintenance does not necessarily equate to the exclusion of minority groups. Rather, its impact on intergroup dynamics depends on how the majority group conceptualizes national identity. If national identity is understood in civic terms—emphasizing shared democratic values and equal participation—cultural maintenance can coexist with inclusiveness (Lefringhausen et al., 2021). Conversely, when national identity is framed in ethnic terms, cultural maintenance can take an exclusionary form by reinforcing the dominance of the mainstream group and limiting national belonging to those who share its heritage (McLaren, 2017). Another distinctive feature of mainstream group acculturation is that, unlike members of minority groups who usually interact with one dominant mainstream group, dominant group members interact with many different minority groups—(Berry, 2017; Kunst et al., 2021). Over time, these cultural exchanges can shift dominant cultural norms and lead to more inclusive views of national identity. By adopting inclusive acculturation strategies, mainstream members demonstrate openness to multicultural integration and may view national identity as expansive enough to incorporate diverse cultural elements (Kunst et al., 2023). Conversely, an exclusive acculturation orientation may reflect resistance to change and emphasize the dominant culture (Lefringhausen et al., 2023). This inclusiveness–exclusiveness spectrum of acculturation orientations aligns with national identity views: inclusive views of national identity promote a sense of common national belonging across diverse groups, whereas exclusive views narrow the national identity to reflect only the mainstream culture. Thus, we conceptualize mainstream people’s national identity inclusiveness as a key indicator of majority group acculturation.

## 5. Psychological Barriers to National Identity Inclusiveness

Adopting an inclusive view of national identity can be particularly challenging for members of the mainstream group, as national identity is deeply tied to emotions, psychological attachment, and perceptions of cultural continuity (Ardag et al., 2019; Eiranen, 2022). For many, national identity is not simply a cognitive category but an emotional investment, closely linked to personal and collective history. As such, shifts toward inclusivity can provoke discomfort, as they may be perceived as a threat to the dominance and distinctiveness of the mainstream group (Craig & Richeson, 2014; Sindic & Condor, 2014). This discomfort is often expressed through exclusionary attitudes and resistance to redefining national identity in more inclusive terms.

Several psychological and sociocultural mechanisms contribute to this resistance. First, cultural identification strength—the degree to which individuals feel attached to their national identity—plays a crucial role. Those who strongly identify with a national identity framed in ethnic terms (e.g., tied to ancestry and cultural heritage such as English-Canadian identity) tend to view inclusivity as a dilution of their group’s cultural dominance, whereas those who identify with a civic conception of national identity (e.g., based on shared values and participation such as multicultural Canadian identity) are more likely to support multicultural inclusion (Yogeeswaran & Dasgupta, 2015; Kunst et al., 2021). Second, identity inclusion–exclusion reflects how individuals categorize others as belonging (or not) to the national ingroup. When national identity is narrowly defined, members of historically dominant groups may struggle to see minorities as legitimate members of the national community, reinforcing exclusionary tendencies (Pehrson & Green, 2010). Finally, acculturation orientations shape attitudes toward diversity. Those who endorse assimilationist views—believing that minorities should conform to mainstream cultural norms—are less willing to expand national identity to accommodate diversity. In contrast, those with integrationist perspectives, which recognize the coexistence of multiple cultural identities within the national identity, are more supportive of inclusive national identity conceptions (Berry, 1997; Verkuyten, 2018).

Thus, changing the perception of national identity to make it more inclusive requires not only intellectual openness but also emotional resilience, as it may challenge long-held beliefs and provoke fear of cultural displacement. Consequently, this study probes a key predictor of both intellectual openness and emotional resilience—cognitive flexibility—as a correlate of national identity inclusiveness.

## 6. Flexible Mind at the Core of Inclusive Views

A plethora of personal and sociopsychological factors have been explored in relation to national or cultural identity (re)configuration, such as intolerance of ambiguity (e.g., Leong & Ward, 2000), openness to experience and extraversion (e.g., Syed, 2013), narcissism (e.g., Bagci et al., 2023), parenting styles (e.g., Vu et al., 2019), multicultural policies, cultural intelligence, and willingness to engage in an intercultural contact (for a review see Nguyen & Benet-Martínez, 2010). Here, we consider another factor that has only attracted researchers’ interest in the field of acculturation and outgroups’ attitudes (Murdock et al., 2013; Chao et al., 2015): cognitive flexibility.

Identity (re)configuration, such as increasing the inclusiveness of one’s national identity representation, is a complex process requiring substantial cognitive resources, especially in the context of superdiversity (Doucerain, 2019; Pekerti, 2019). When negotiating different and sometimes conflicting identity elements, people must exhibit considerable flexibility to reconcile cultural differences and adequately manage automatic responses such as prejudices and stereotypes. Balancing emotional reactivity and rational thought is crucial in this process, as it ensures that people can navigate these complex dynamics without being overwhelmed by emotions, thus making more informed and thoughtful decisions.

M. M. Martin and Rubin (1995) define cognitive flexibility as the ability to adjust one’s thinking or behavior in response to changing situational demands. This involves recognizing the need for change, the ability to generate alternative strategies, and implementing these strategies effectively. Cognitive flexibility encompasses skills such as shifting perspectives, adapting to new information, and fluidly transitioning between different tasks or mental frameworks. Cognitive flexibility is associated with the ability to freely shift thoughts or behavior in response to changing task or situational demands, develop diverse ideas, consider response alternatives, amend plans, and a disposition to be flexible (Rende, 2000). Facets of cognitive flexibility that allow a person to think adaptively in stressful life events are an inclination to characterize challenging situations as controllable, the ability to identify numerous different viewpoints in each situation, the capacity to generate alternative approaches to difficult situations, and the ability to effectively manage emotions (Dennis & Vander Wal, 2010). Such flexibility is crucial for navigating complex, dynamic environments.

Cognitive flexibility is consistent with having a more inclusive national identity representation, in that the tendency to see mutual influences and culture as dynamic requires the capacity and motivation to generate alternative explanations in racially and ethnically diverse settings and to look beyond traditional cultural norms. In the context of intergroup processes, experimental work by Rokeach (1948) concluded that rigidity and inflexibility of the thinking process were associated with ethnocentrism. This was further refined by Adorno (1950), who found evidence to support his hypothesis that prejudice is closely linked to cognitive rigidity and intolerance of ambiguity. Correlates of inflexibility also include conservatism (Van Hiel et al., 2016) and extremism (Zmigrod et al., 2019). According to Crisp and Turner (2011), cognitive flexibility is enhanced when social and cultural diversity is encountered by successfully integrating conflicting frames of reference. Chao et al. (2015) argued that flexibility provides those high on openness to experience with the tools to embrace other cultures and develop adaptive solutions that may facilitate social coordination. More recently, cognitive flexibility has been explored as a factor behind multilingual people being more accepting of ethnic outgroups (Mepham & Martinovic, 2018). In a similar vein, it has been associated with more complex cultural identities among both majority and minority members (Medvetskaya et al., under review). Cognitive flexibility likely shapes the intricate processes of identity negotiation and reconfiguration, fostering more inclusive national identity representations and, in turn, influencing majority group acculturation more broadly.

## 7. The Present Study

The current study explores patterns of national identity views among mainstream cultural group members in Canada, focusing on the inclusiveness of these views. Please note that we included all Canadian provinces except Quebec. Quebec was excluded due to its unique cultural and linguistic identity, which may introduce distinct patterns of national identification that differ from the rest of Canada (e.g., Taylor, 1991; Mendelsohn, 2002). While Canada’s national identity is built on the recognition of cultural diversity as a core value (Ryan, 2010), Quebec positions itself as a distinct nation, using its unique cultural identity to strengthen its arguments for independence (Paquin, 2018). This exclusion criterion allows for a more focused investigation of national identity within the broader Canadian context, reducing potential confounding variables. 

Canada was selected as the setting for this study due to its distinctive approach to multiculturalism, which is not only a social reality but also an official policy. Since the introduction of the Multiculturalism Policy in 1971, Canada has positioned itself as a model for managing cultural diversity, theoretically promoting an inclusive view of national identity that embraces diverse ethnic and cultural backgrounds (Kymlicka, 1998). Canada is thus well suited for examining how national identity is conceptualized among mainstream members, particularly in terms of its inclusiveness. Multiculturalism is officially promoted, but it is crucial to examine how this policy is reflected in the national identity representations of the historically dominant group—White English-speaking Canadians. Since European settlement, this group has historically shaped the national narrative and may endorse a more exclusive view of national identity, emphasizing traditional and homogeneous aspects of Canadian identity^2^. By focusing on this demographic, the study seeks to uncover potential tensions between official national identity representations as promoted by multicultural policies and the lived experiences and attitudes of the mainstream cultural group in Canada. 

To analyze the data, we employed latent class regression analysis (LCRA), a statistical method that identifies unobserved subgroups within a population based on participants’ responses. LCRA was chosen for its ability to categorize people into distinct latent classes based on their views on national identity, rather than forcing a binary or linear categorization. This method allows for the capture of the complexity of national identity views by identifying patterns of beliefs that may not be immediately apparent through traditional variable-based analytical techniques (Collins & Lanza, 2009). In cultural psychology, LCRA can reveal distinct cultural profiles and acculturation strategies, providing deeper insights into how people navigate multiple cultural contexts and integrate cultural identities (Bámaca-Colbert & Gayles, 2010; Huang, 2005). LCRA accommodates the complexity and heterogeneity inherent in cultural experiences, moving beyond simplistic, single-dimensional classifications. It also helps in understanding the nuanced relationships between cultural identities, behaviors, and psychological outcomes.

This study examines distinct patterns of national identity views—whether they are more inclusive or exclusive, and investigates cognitive flexibility, a hitherto unexamined correlate of belonging to each identified pattern. Age and gender were also included as covariates. Consistent with earlier research linking cognitive flexibility to openness to different perspectives (M. M. Martin & Rubin, 1995), we hypothesized that higher levels of cognitive flexibility would be associated with a greater likelihood of belonging to a class characterized by a more inclusive view of national identity.

## 8. Method

### 8.1. Participants and Procedure

Participants were recruited using Prolific Academic, an online platform for participant recruitment. All were adults residing in Canada. The inclusion criteria were to be at least 18 years old, to be born in Canada, to self-identify as White, and to have English as the first language. Participants were asked to complete an online survey on Qualtrics. All participants provided informed consent and were compensated for their participation in accordance with Prolific Academic’s guidelines. Median completion time was 25 min. The ethics review board of the authors’ universities approved the study protocol.

Our initial sample comprised 219 participants. We kept only those who had at least one parent born in Canada to maintain the cultural homogeneity of the mainstream group. The final sample included 202 participants (64.85% self-identified as women, 32.67% as men, 2.47% as another gender identity), with a mean age of 34.89 years (*SD* = 13, range 18–75 years). The majority of the sample (86.63%) had both parents born in Canada. Almost 64% reported working either full-time or part-time, 19.8% were students, 6.9% were unemployed, 3.5% reported being retired, and 1.5% had a disability status not allowing them to work. Slightly more than half of the participants (53.4%) had at least an undergraduate degree. These statistics diverged from those of the corresponding group of the general population in the national census (50.37% men, 49.63 women, about 78% of respondents are employed, about 7% are students, and about 27% hold at least an undergraduate degree, Statistics Canada, 2024), skewing our data in terms of gender representation and education. While corresponding to the inclusion criteria for the mainstream group, 21.8% of the participants self-identified as multicultural; others self-identified as monocultural.

### 8.2. Materials

To capture the inclusiveness of national identity, we used three sets of measures: cultural identification strength, identity inclusion–exclusion, and acculturation orientations. The first measure captures the depth of one’s attachment to their cultural group, reflecting how central this group is to their identity. The second measure evaluates the extent to which one’s perceptions of the Canadian identity are inclusive or exclusive. In this study, orientations toward multicultural Canadian identity and toward heritage English Canadian identity are used as proxies for inclusive and exclusive views on Canadian identity. Finally, acculturation orientations measure assesses dispositions of the mainstream group members toward both maintaining their culture and adopting the cultural practices of other groups. Combining these measures provides a comprehensive assessment of Canadian identity representations, grounded in the common ingroup identity, the ingroup projection models, and the acculturation model. Further, collectively, these measures allow for the assessment of both cognitive and affective dimensions of identity. Strength of cultural identification provides insight into the affective component—how emotionally attached people are to their cultural group. In contrast, the Canadian inclusion–exclusion measure captures the cognitive aspect—how people rationalize the inclusivity or exclusivity of Canadian identity. Acculturation orientations, in their turn, address cognitive and emotional components—such as beliefs, values, and attitudes regarding cultural maintenance and participation.

#### 8.2.1. Cultural Identification Strength

The Inclusion of Ingroup in the Self scale (IIS; Tropp & Wright, 2001) assesses how much a social/cultural group is included in the self. The scale consists of 7 pairs of circles with varying degrees of overlap, from none (1) to almost complete (7). Participants select the pair that best reflects their personal identification level. We used two items to measure personal identification with the English Canadian identity (En Ca ID) and a multicultural/diverse Canadian identity (Multi Ca ID).

#### 8.2.2. Canadian Identity Inclusion

Inclusiveness and exclusiveness of American identity scale (InExIS; Doucerain et al., 2018) was modified to include ten items assessing to what extent participants perceive the Canadian identity as inclusive or exclusive (“In your opinion, to what extent are the groups below representative of the Canadian identity?”). Participants rated these items on a 7-point Likert scale (1 = do not agree at all, 7 = agree very strongly). The Inclusive Identity subscale (Inclusive Ca ID) included: “The various ethnic groups that live in Canada”, “The various linguistic groups that live in Canada”, “The diverse cultural groups that compose the country”, “All of Canada’s diverse population”, and “All of Canada’s melting pot of citizens”. The Exclusive Identity subscale (Exclusive Ca ID) included: “Only groups of people who are of European descent”, “Groups of people who share similar cultural characteristics with the first settlers in Canada”, “Only those who are native speakers of English”, “Group members who embrace core Canadian values (such as equality, freedom, etc.)”, and “Only people who were born in Canada”. The reliability coefficients were α = 0.91 for the inclusion subscale and α = 0.77 for the exclusion subscale.

#### 8.2.3. Majority Acculturation Orientations

The Brief Acculturation Orientation Scale (BAOS; Demes & Geeraert, 2014) measures orientations toward heritage and mainstream cultural contexts, valuing cultural friendships, traditions, characteristics, and behaviors. Participants rate four statements per subscale on a 7-point Likert scale (1 = strongly disagree, 7 = strongly agree). In our study, we adapted the scale to the mainstream population to reflect their English Canadian and multicultural/diverse Canadian cultural orientations. Examples are, “It is important for me to hold on to my English Canadian characteristics”, and “It is important for me to have multicultural friends”, respectively. Both subscales showed excellent reliability: Cronbach’s α = 0.91 for heritage English Canadian orientation (BAOS-H) and α = 0.91 for mainstream multicultural orientation (BAOS-M).

#### 8.2.4. Cognitive Flexibility

The Cognitive Flexibility Scale (CFS; M. M. Martin & Rubin, 1995) is a 12-item self-report measure assessing cognitive flexibility. This concept involves recognizing communication options in any situation, willingness to adapt, and self-efficacy in being flexible. Sample items include “I can communicate an idea in many different ways” and “I can find workable solutions to seemingly unsolvable problems.” Responses are recorded on a 6-point Likert scale (1 = strongly disagree, 6 = strongly agree), with four items reverse-scored.

### 8.3. Analysis

All statistical tests were conducted with a significance level set at 0.05 (95% confidence), unless otherwise stated. First, the data were inspected for univariate and multivariate outliers, normality, and missing values. We found univariate outliers for identification with mainstream multicultural/diverse Canadian identity (1 value, 0.49%). We winsorized them by bringing extreme values outside three median absolute deviations around the median within that interval (Leys et al., 2013). No multivariate outliers were detected based on Mahalanobis distances evaluated at *p* < 0.001. None of the variables exhibited skewness or kurtosis with absolute values greater than 1, and QQ-plots showed no significant deviations. Additionally, no variable had more than 5% of the missing data. Subscales for inclusivity and exclusivity of Canadian identity both had three missing values (1.48%). We investigated the patterns of missing data through visual analysis and by employing the Projected Kullback–Leibler MCAR test (Spohn et al., 2021), a non-parametric approach for evaluating the missing completely at random (MCAR) assumption. We found the data to be MCAR at *p* = 0.078.

Latent class regression analysis (LCRA) was used for the main analysis, with the R (R Core Team, 2024) package poLCA (Linzer & Lewis, 2011). Power estimation for latent class analysis (LCA) is affected by multiple factors influencing the distribution of model parameters, such as the number of estimated classes, class separation, covariate quality, and the reliability of within-class observations (Dziak et al., 2014; Jaki et al., 2018). Generally, a sample size of around 500 is recommended (Nylund et al., 2007). However, sample sizes comparable to ours have been used in similar studies (e.g., *n* = 230 in Driscoll & Torres, 2022; *n* = 319 in Bámaca-Colbert & Gayles, 2010). Though larger sample sizes are generally preferred in latent class analysis (LCA) to ensure robust and reliable results, smaller sample sizes can sometimes be sufficient if the indicators used are of high quality. In such cases, the quality of the indicators can help mitigate the challenges associated with a smaller sample. To assess the quality of the indicators in our model, multiple checks were conducted. First, classification uncertainty was evaluated, yielding a value of 0.07, which indicates low uncertainty and suggests that individuals are being assigned to latent classes with high confidence (Clark & Muthén, 2009). Second, visual inspection of the uncertainty distribution showed values clustering near zero, reinforcing the reliability of class assignments. Third, a box plot of posterior probabilities demonstrated strong class separation. These checks suggest that the indicators were of high quality, as they effectively distinguish between latent classes. High-quality indicators reduce classification error and improve model interpretability (Collins & Lanza, 2009) and can be even more beneficial for smaller sample sizes (Wurpts & Geiser, 2014).

We conducted latent class regression analysis with class indicators and predictors of class membership all included in a single model. Indicators included cultural identification strength, the inclusiveness and exclusiveness of Canadian identity, and English Canadian and acculturation orientations. Predictors were gender, age, and cognitive flexibility. Models with progressively more classes (two to four) were tested using the R package poLCA (Linzer & Lewis, 2011) and with maximum likelihood estimation. To statistically assess model fit, we used BIC and AIC (smallest values), relative entropy (>0.70), size of the smallest class (at least 10%), and posterior probabilities (mean probability >0.70). Table 1 displays goodness of fit statistics. All models had adequate statistical fit, and the bootstrap likelihood ratio test indicated no significant differences between solutions that had more than 2 classes (*p* = 0.33). Thus, based on the bootstrap likelihood ratio test, lowest BIC, almost uniform distribution of predicted class memberships (52.97%; 47.03%), and theoretical interpretability of classes, we chose the 2-class model (Morgan, 2015; Nylund-Gibson & Choi, 2018).

In LCA, for assessing model fit, the preference is often given to BIC as it balances model fit and complexity, but, unlike AIC, also imposes a stricter penalty for complex models. The BIC index favors more parsimonious models, which makes the results more interpretable and practical for researchers (Vermunt & Magidson, 2002; Yang, 2006). The four-class model was rejected despite better relative entropy, comparable mean posterior probability, and lower AIC because of the smallest class size (<10%) and for theoretical reasons. Models with too many classes can become difficult to interpret, and their choice may indicate that models are being driven by statistical convenience rather than meaningful distinctions in the data. Each class should represent a distinct, meaningful subgroup within the data. With only six indicators, the differences between classes may be subtle and not substantial enough to justify separate classes, making the model complex and less useful for practical purposes. Additionally, the results of the bootstrap likelihood ratio test (LRT) supported the rejection of the three-class solution, further reinforcing our decision to opt for a two-class model based on both statistical and theoretical considerations.

## 9. Results

### 9.1. Descriptive Results

Table 2 shows descriptive statistics and Spearman correlations among continuous variables. On average, participants endorsed a relatively strong identification with the English Canadian cultural group (*M* = 3.93, *SD* = 0.97), indicating a moderate to high level of alignment with this mainstream identity. Multicultural identification was endorsed at a moderate level (*M* = 2.83, *SD* = 0.97), suggesting participants recognize diverse cultural perspectives, but with less emphasis than on the mainstream Canadian identity. Regarding Canadian identity inclusiveness, participants showed strong support for a more inclusive vision of Canadian identity (*M* = 4.90, *SD* = 1.43), reflecting a favorable stance toward incorporating diverse cultural backgrounds. On the other hand, the endorsement of Canadian identity exclusiveness was moderate (*M* = 3.21, *SD* = 1.30), indicating a slight preference for a more traditional, homogeneous national identity. Regarding acculturation orientations, participants demonstrated a moderate inclination toward maintaining their cultural heritage (*M* = 4.02, *SD* = 1.56) and showed a relatively strong preference for multicultural engagement (*M* = 4.15, *SD* = 1.52).

The paired *t*-tests using the same sample showed that, overall, participants scored higher on English Canadian identification than on multicultural identification (*t*(201) = 11.09, *p* < 0.001) and Canadian identity inclusiveness than exclusiveness (*t*(201) = 10.59, *p* < 0.001). At the same time, there was no significant difference between mean scores on cultural orientations toward English Canadian and multicultural streams (*t*(201) = 0.95, *p* = 0.34). On average, participants demonstrated quite high cognitive flexibility (*M* = 4.98, *SD* = 0.81).

English Canadian personal identification was positively correlated with the perception of Canadian national identity as exclusive and with the cultural orientation toward the heritage English Canadian cultural stream. Multicultural personal identification, in turn, was positively correlated with the inclusiveness of Canadian national identity and with the cultural orientation toward multicultural identification. At the same time, Canadian national identity inclusiveness and exclusiveness showed a negative correlation of moderate strength, supporting the idea that these perceptions reflect separate dimensions (Doucerain et al., 2018; Mummendey & Otten, 1998). Both measures correlated with the measures of cultural orientation: inclusiveness was positively related to the orientation toward multicultural stream and negatively to the heritage English Canadian cultural stream, exclusiveness showed the opposite pattern. Both cognitive flexibility and orientation toward heritage English Canadian cultural stream were positively related to age.

### 9.2. Latent Class Description and Regressions

**Figure 1:**
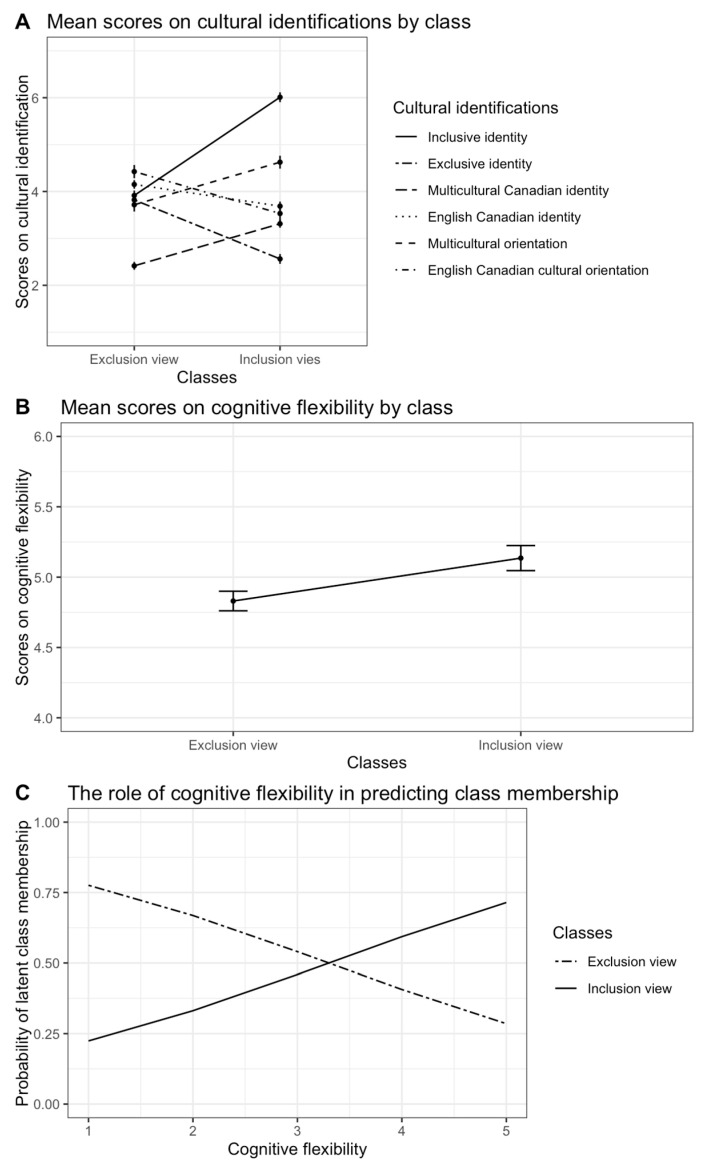
Mean scores and covariate effects on class belonging probability. *Note.* Error bars represent ±1 standard error of the mean.

Based on the maximum allocation probability, participants were assigned to classes that comprised 52.13% and 47.87% of the sample. Figure 1A illustrates the patterns of responses in inclusion–exclusion views on cultural identification. The probability to endorse greater scores on multicultural acculturation orientation, multicultural personal identification, and the inclusive view of the Canadian national identity was higher in the first class (mean score difference between classes *t*(199.54) = 5.44, *p* < 0.001 for multicultural acculturation orientation; *t*(198.63) = 7.82, *p* < 0.001 multicultural personal identification; *t*(198.56) = 12.88, *p* < 0.001 for Canadian identity inclusiveness). This class was labeled as *inclusion view*. In turn, the second class had the highest probabilities of scoring high on English Canadian acculturation orientation, English Canadian personal identification, and Canadian identity exclusivity (mean score difference between classes *t*(188.9) = 2.93, *p* = 0.004 for English Canadian acculturation orientation; *t*(194.66) = 3.58, *p* < 0.001 for English Canadian personal identification; *t*(199.14) = 7.46, *p* < 0.001 for Canadian identity exclusivity), and was labeled *exclusion view*. Interestingly, as Figure 1A demonstrates, English Canadian personal identification was higher than multicultural personal identification in both *inclusion view* (*t*(106) = −3.66, *p* < 0.001) and *exclusion view* (*t*(94) = 14.79, *p* < 0.001) classes. However, it was significantly lower in the *inclusion view* class than in the *exclusion view* class (*t*(194.66) = −3.58, *p* < 0.001), suggesting a decreasing tendency in the strength of personal identification with the English Canadian group in the presence of increasing strength of identification with the multicultural group. As expected, mean score on cognitive flexibility was higher in the *inclusion view* class (*t*(182.5) = 2.72, *p* = 0.007, see Figure 1B).

We assessed the effects of predictors on the probabilities of belonging to the inclusion view class versus the exclusion view class. Table 3 summarizes these results in the form of odds ratios. Consistent with our expectation, cognitive flexibility increased chances to belong to the *inclusion view* class (1.77 times more chances, *B* = 0.57, *SE* = 0.27, *p* = 0.037). Figure 1C illustrates this striking effect of cognitive flexibility on the probability of belonging to the *inclusion view* class compared to the *exclusion view* class. Neither age nor gender had a significant effect on the belonging probability.

We also conducted additional sensitivity analyses, available as Supplementary Materials. Sensitivity analyses were conducted by running the model separately for participants with two Canada-born parents, those who self-identified as monocultural, and those whose parents had English as their first language (suggesting a closer alignment with the dominant cultural group, as language is a crucial component of social integration and identity). The results for all three subsample analyses reveal a similar pattern of exclusion vs. inclusion views. In terms of the role of cognitive flexibility, the regression coefficient was statistically significant for the analysis with participants whose parents spoke English and marginally significant for the analysis with participants who self-identified as monocultural. In both cases, greater cognitive flexibility was associated with a greater likelihood of belonging to the class with more inclusive views of national identity.

## 10. Discussion

This study explored exclusive and inclusive views of national identity among majority group members on the individual level using latent class regression analysis. We probed the role of an individual variable—cognitive flexibility—in predicting patterns of responses on inclusive or exclusive national identity, conceptualized as a key aspect of majority group acculturation. We expected that viewing national identity as multicultural, along with a stronger attachment to and orientation toward multicultural groups, would correspond with more inclusive perspectives on Canadian identity, and that this pattern of responses would be associated with a higher score on cognitive flexibility. The results discussed below supported our hypothesis.

Borrowing from theoretical models of group inclusion—common ingroup identity, ingroup projection, and majority group acculturation models—we assessed the inclusiveness of national identity on different levels: at the level of conceptual representation of a superordinate identity, at the level of personal identification, and at the level of personal attitudes toward national identity inclusiveness. Conceptualizing the national identity as multicultural, stronger identification with multicultural Canadian identity versus English Canadian identity, and orientations toward sharing this identity with others, all aligned in a pattern of responses characterized by more inclusive views on national identity. This suggests that people from the mainstream cultural group can view diverse groups as part of a larger, shared national identity, promoting unity and reducing intergroup bias, and are less likely to project their own cultural norms onto the national identity, demonstrating a greater openness to diverse cultural contributions within the national framework. 

Importantly, such openness does not contradict their positive attitudes toward the English Canadian heritage cultural group. As Figure 1A demonstrates, people from the mainstream cultural group may strongly identify with their cultural heritage and yet be open toward the inclusion of others in their sense of national identity. This finding is in line with the traditional acculturation model, highlighting the importance of simultaneous acculturation orientations toward heritage and mainstream cultures (Berry, 1997, 2003; L. Martin et al., 2019; Robertson & Grant, 2023; Van de Vijver et al., 1999; Van Oudenhoven et al., 1998; Yu & Wang, 2011).

Though acculturation orientations unfold differently in the majority group, reflecting their orientations toward inclusion or exclusion of others rather than a desire to adapt to other cultures, this finding underlies the same chief tendency of being able to simultaneously orient toward different cultural streams. Importantly, it highlights the significance of considering inclusiveness tendencies not only at the level of conceptualization of national identity, but also at the level of individual identification. Some mainstream group members may be inclined to view national identity as multicultural because it aligns with a progressive self-image. In societies marked by super-diversity like Canada, Australia, or the UK, promoting diversity is often seen as a marker of modernity and inclusivity (Guan & Pietsch, 2024; Wildeman, 2021). Diversity policies allow majority group members to position themselves as open-minded (Landemore, 2018). This framing of cultural diversity offers a way to reflect their nation’s progressive values, often fostering a sense of moral superiority or international prestige.

As Modood (2024) explains, multicultural national identity involves incorporating minority identities into a broader national framework, which helps ease identity-related anxieties of both minority and majority groups while promoting a more cohesive society. By supporting cultural diversity, mainstream groups can demonstrate their commitment to egalitarian values without necessarily relinquishing their cultural dominance. Such inclination, however, may be at odds with how people from the mainstream cultural group actually *feel* about their own belonging. A sense of belonging would be inevitably rooted in everyday social and cultural environments—beyond formal institutions—where one feels “at home” (Alba, 2009). However, feeling “at home” means not just familiarity and safety, but also a sense of control and authority over who belongs (Yogeeswaran et al., 2011). Thus, it is possible for a mainstream individual to endorse an inclusive view on national identity while personally identifying strongly with her cultural heritage. Capturing responses on multiple dimensions of national identification at the individual level allows for the identification of emerging patterns of inclusive views that reflect a stronger capacity for inclusiveness, where national identity is seen as flexible and capable of incorporating diverse cultural perspectives without being constrained by majority norms and values.

Our second important finding sheds light on the role of cognitive flexibility in the process of constructing national identity. Cognitive flexibility refers to the mental ability to adapt and shift perspectives and adjust emotional responses, which is essential when considering diverse cultural backgrounds. Scoring high on this variable was associated with a higher probability of endorsing a more inclusive view on national identity. This suggests that people who are more cognitively flexible may be better equipped to adjust their conceptualization of national identity to include diverse cultural groups. This adaptability allows them to challenge rigid or exclusionary views of identity, fostering a more inclusive perspective.

Though research on cognitive flexibility as a variable implicated in (re)configuration of national identity as more inclusive is almost non-existent, some previous studies support the idea that cognitive flexibility may enhance inclusive views on others. For example, Sparkman et al. (2019) have found that the ability to take perspectives of other ethnic groups mediated the relation between personality traits and openness to diversity/support for multiculturalism among Americans (85% of whom were White). Bernardo and Presbitero (2018) hypothesized that cognitive flexibility might be implicated in cultural competence, which reflects the effective functioning in diverse environments. They found that the dimension of cognitive flexibility related to the ability to shift perspectives was positively related to cultural competence in Chinese students in Macau. Further, reflecting the role of cognitive flexibility in emotional regulation, the findings of Liu and Wei (2020) demonstrated that cognitive flexibility was positively associated with more empathy toward other cultures, mitigating the effect of acculturation stress among Chinese international students in the US.

Possibly, cognitive flexibility is a key variable that could explain what enables people to navigate the complexities of cultural pluralism and reconcile potential conflicts between their cultural heritage and the broader national identity. Cognitive flexibility, encompassing executive functions such as perspective-taking, decision-making, and emotion regulation, can serve as a mediator between stable personality traits like openness to experience and final thought products (Mepham & Martinovic, 2018). It may do so by allowing people to process conflicting information, reconsider their assumptions, and manage emotional reactions, leading to more open-minded and balanced conclusions (Doucerain, 2019). Considering cognitive flexibility allows a better understanding of how some people manage to expand their notions of national identity, while others remain resistant to inclusiveness. Thus, cognitive flexibility may be an important factor to consider in our understanding of the majority group acculturation.

## 11. Conclusions

Inclusiveness of national identity and broader inclusive views are of critical importance in today’s globalized world, as societies are becoming increasingly diverse. Ensuring inclusivity promotes social cohesion, reduces intergroup conflict, and strengthens a sense of belonging among all citizens, which is vital for fostering unity in multicultural environments. This research assessed how perceptions about national identity, ingroup identification, and orientations toward inclusion of others align to form a representation of inclusive national identity at the individual level and how cognitive flexibility might be related to endorsing more inclusive views. Our results suggest that cognitive flexibility should be an important consideration in research on identity inclusiveness and mainstream group acculturation for several reasons. Traditional pre-constructed measures of inclusiveness often “assign” categories developed by researchers to participants, capturing the outcome but not the underlying reasons for their choices. Taken alone, these measures fail to explain why certain people may lean toward specific inclusive or exclusive views. Cognitive flexibility, as a mental capacity for adapting to new perspectives and managing conflicting information, may be a key psychological factor that underlies these inclinations. By focusing on cognitive flexibility, researchers may better understand the deeper cognitive processes involved in how people approach national identity, moving beyond descriptive categories.

Furthermore, evidence suggests that cognitive flexibility can be developed through targeted interventions, which holds direct implications for fostering more inclusive attitudes (Braem & Egner, 2018; Glass et al., 2013). Training people to enhance their cognitive flexibility could potentially broaden their perspectives and increase their openness to diversity—important outcomes in today’s increasingly multicultural societies. Therefore, integrating cognitive flexibility into research on national identity inclusiveness can not only provide a deeper understanding of individual differences in inclusiveness but also point to practical strategies for promoting inclusivity in diverse environments.

### Limitations and Future Directions

The present study provided new insights into how the majority group members view national identity inclusiveness at an individual level, highlighting its connection to higher cognitive flexibility. When considering the role of cognitive flexibility as a psychological mechanism supporting inclusiveness, it is important to take into account the limitations of this study. One such limitation is the sample, which consisted solely of Prolific Academic participants. While Prolific offers access to a large and diverse pool of participants, those who participate in academic research on this platform often possess higher levels of education, intellectual engagement, and familiarity with research processes, factors that could skew results. Additionally, participants on Prolific may self-select based on an interest in or predisposition toward social science topics, further limiting representativeness. Further, our sample may not be fully representative of the broader Canadian population, given the overrepresentation of women compared to men and the educational background of participants, which limits the generalizability of the findings. As a result, findings from this sample may not fully capture the link between inclusiveness views and cognitive flexibility levels of the broader population, particularly those with lower educational attainment or less exposure to diverse perspectives. Future studies should consider including participants from varied educational, socioeconomic, and cultural backgrounds to provide a more comprehensive understanding of how cognitive flexibility influences inclusivity in national identity. Moreover, this study focused solely on English-speaking participants. Including other groups, such as French-speaking Canadians or various minority populations, would provide a more comprehensive analysis and broaden the generalizability of the results.

Another major limitation was the relatively small sample size used in the study, which may impact the robustness of the latent class analysis. This analysis typically benefits from larger datasets, and future research with larger samples would help strengthen the generalizability of the findings. The findings from the quality of indicators assessment support the use of the relatively small sample size in our analysis, as robust class differentiation can still be achieved with fewer participants when indicators are strong (Nylund-Gibson & Choi, 2018). However, power considerations in future studies should still be evaluated to ensure adequate sensitivity for detecting effects of covariates. Also, while latent class analysis is widely recognized in the field of cross-cultural and social psychology, future research may look at alternative statistical methods that are particularly useful for handling complex, non-linear relationships and can offer more nuanced insights into data structure. For instance, fuzzy logic and fuzzy clustering can capture degrees of membership across categories, making them well suited for studying ambiguous or overlapping group membership (Bezdek, 1981). Similarly, quantile regression can provide a deeper understanding of relationships at different points in the distribution of the dependent variable (Koenker, 2005).

Age and gender were controlled for due to their established influence on national identity perceptions; however, the exclusion of other potentially influential variables, such as socioeconomic status or levels of prejudice, represents a limitation, and future research should account for their potential impact. Additionally, while alternative predictors such as perceived prototypicality and in-group distinctiveness threat have been discussed in the literature, their absence from the analyses warrants acknowledgment, and their inclusion in future studies could provide a more comprehensive understanding of the factors shaping national identity inclusiveness.

Furthermore, our study relied exclusively on self-report measures to assess cognitive flexibility. However, previous research has shown that self-reported measures of cognitive flexibility often do not align well with task-based measures of cognitive flexibility that capture specific components of executive functioning, such as switching between tasks (Johnco et al., 2014). To address this limitation, future studies should incorporate a wider range of cognitive flexibility assessments, including task-based or observational methods, to provide a more comprehensive understanding of psychological mechanisms implicated in identity configuration. It is also important to note that the cognitive flexibility measures used in this study were not explicitly designed to assess responses to culturally diverse or intergroup situations. Despite this, the observed relationship between cognitive flexibility and more inclusive views on national identity suggests the involvement of deeper psychological processes that contribute to the development of inclusiveness, which may extend beyond specific social or cultural contexts.

## Figures and Tables

**Table 1:** Model Fit of Latent Class Regression Analyses With Up to Four Latent Classes (*N* = 202).

	Number of Latent Classes		
	2	3	4
Size of the smallest class, %	42.87	21.18	7.89
Maximum Log-Likelihood	−1871.83	−1826.46	−1795.34
No. of Estimated Parameters	68	104	140
AIC ^a^	3879.67	3860.92	3870.67
BIC ^a^	4104.63	4204.98	4333.83
Mean posterior probability	0.93	0.94	0.91
Relative Entropy	0.76	0.85	0.84
Bootstrap LRT, *p* Value ^b, c^	–	0.33	0.35

*Note.* ^a^ AIC, Akaike’s information criterion; BIC, Bayesian information criterion; LRT, likelihood ratio test. Incremental changes in BIC < 2 are considered marginal (Kass & Raftery, 1995, p. 777). ^b^ LRT, likelihood ratio test according to Nylund et al. (2007). ^c^ If *p* < 0.05, it will fit significantly better than a model with (*k* − 1) latent classes.

**Table 2:** Means, standard deviations, and correlations with confidence intervals.

Variable	*M (SD)*	1	2	3	4	5	6	7
1. Age	34.89 (13.00)							
2. English Canadian identification	3.93 (0.97)	0.12						
		[−0.02, 0.25]						
3. Multicultural identification	2.83 (0.97)	−0.04	−0.05					
		[−0.18, 0.10]	[−0.18, 0.09]					
4. Canadian ID inclusiveness	4.90 (1.43)	−0.11	−0.11	0.34 **				
		[−0.25, 0.03]	[−0.24, 0.03]	[0.21, 0.45]				
5. Canadian ID exclusiveness	3.21 (1.30)	0.02	0.14 *	−0.11	−0.39 **			
		[−0.12, 0.16]	[0.00, 0.28]	[−0.25, 0.03]	[−0.50, −0.27]			
6. BAOS-H	4.02 (1.56)	0.15 *	0.27 **	0.05	−0.15 *	0.27 **		
		[0.01, 0.28]	[0.14, 0.40]	[−0.09, 0.19]	[−0.29, −0.02]	[0.14, 0.40]		
7. BAOS-M	4.15 (1.52)	−0.13	−0.03	0.48 **	0.25 **	−0.15 *	0.16 *	
		[−0.26, 0.01]	[−0.17, 0.11]	[0.36, 0.58]	[0.11, 0.37]	[−0.28, −0.01]	[0.02, 0.29]	
8. Cognitive flexibility	4.98 (0.81)	0.23 **	−0.04	0.13	0.1	0.07	0.05	0.06
		[0.09, 0.35]	[−0.18, 0.10]	[−0.00, 0.27]	[−0.04, 0.24]	[−0.07, 0.20]	[−0.09, 0.18]	[−0.08, 0.19]

*Note.* Statistical significance is marked by * *p* < 0.05 and ** *p* < 0.01. BAOS-H: English Canadian heritage cultural orientation, BAOS-M: Multicultural/diverse Canadian cultural orientation. Values in square brackets indicate the 95% confidence interval for each correlation. The confidence interval is a plausible range of population correlations that could have caused the sample correlation (Cumming, 2014).

**Table 3:** Unstandardized odds ratios for acculturation class membership.

	Inclusion View vs. Exclusion View	
Covariates	OR (SE)	*p*
Gender (female)	1.04 (0.47)	0.24
Age, years	0.97 (0.01)	0.074
CFS	1.77 (0.27)	0.037

*Note*. OR = odds ratios, SE = standard errors. Odds ratios are calculated using unstandardized betas from latent profile regression models as exponents of the natural base e.

## Data Availability

The raw data supporting the conclusions of this article will be made available by the authors on request.

## Notes

1 The acculturation orientations traditionally assessed in migrant populations reflect a need to negotiate the feeling of belonging to at least two cultural groups—their heritage cultural group and the local majority group (Berry, 1997, 2005). As such, it allows four possible acculturation strategies that capture simultaneous engagement and identification with heritage and mainstream cultural groups.

2 It must be noted, however, that this is recent history, rooted in Canada’s colonial past under the UK and France, with First Nations peoples predating European settlement. Although now a small proportion of the population, Indigenous communities have played a key role in shaping national identity, reconciliation efforts, and Canada’s evolving approach to multiculturalism.

## Supplementary Materials

The following supporting information can be downloaded at: https://www.mdpi.com/article/10.3390/bs15040498/s1.
