# Supplementary material for: Thinking Outside the Nation: Cognitive Flexibility’s Role in National Identity Inclusiveness as a Marker of Majority Group Acculturation
Source: Behav Sci (Basel). 2025 Apr 9;15(4):498. doi: 10.3390/bs15040498 (PMC12024263; doi:10.3390/bs15040498)
Supplement: Supplementary file 1 [file behavsci-15-00498-s001.zip › behavsci-3502963-supplementary.pdf]

I. Sensitivity analysis for different subsamples

1. Subsample of participants having both parents born in Canada (N=175)

```
Estimated class population shares
 0.53; 0.47

=========================================================
Regression coefficients:
=========================================================
2 / 1
              Coefficient Std. error t value  Pr(>|t|)
(Intercept)   -1.23638     1.52275   -0.812     0.419
sexFemale     -0.05880     0.51446   -0.114     0.909
age           -0.02478     0.01681   -1.474     0.144
cfs            0.40444     0.27143    1.490     0.139
=========================================================

number of observations: 175
number of estimated parameters: 70
residual degrees of freedom: 105
maximum log-likelihood: -1621.288

AIC(2): 3382.577
BIC(2): 3604.112
X^2(2): 214314.8 (Chi-square goodness of fit)

Mean posterior probabilities
Class 1         0.94
Class 2         0.92
Across All Classes       0.93

Relative entropy  0.79
```

Visualisation of response patterns by class

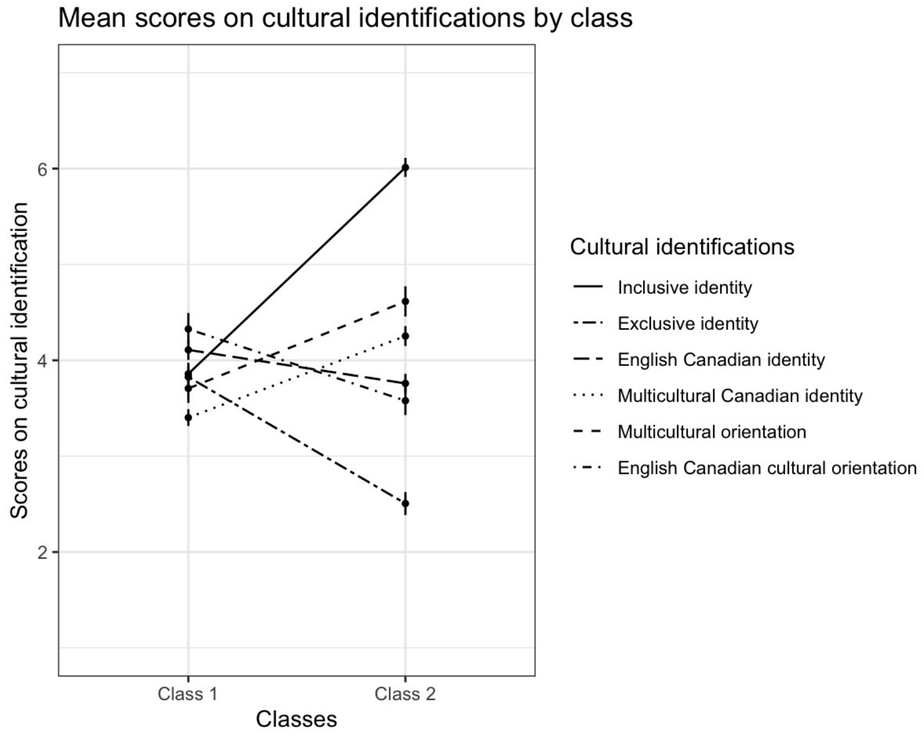

2. Subsample of participants who self-identified as monocultural (N=158)

```
Estimated class population shares
 0.60; 0.40

=========================================================
Regression coefficients:
=========================================================
2 / 1
              Coefficient  Std. error  t value  Pr(>|t|)
(Intercept)    -2.13765     1.76127   -1.214     0.228
sexFemale       0.07787     0.66127    0.118     0.907
age            -0.03705     0.02135   -1.735     0.086
cfs             0.58781     0.30864    1.905     0.060
=========================================================

number of observations: 158
number of estimated parameters: 72
residual degrees of freedom: 86
maximum log-likelihood: -1461.233

AIC(2): 3066.465
BIC(2): 3286.972
X^2(2): 274797.4 (Chi-square goodness of fit)

Mean posterior probabilities
Class    1         0.95
Class    2         0.92
Across All Classes   0.94

Relative entropy 0.79
```

Visualisation of response patterns by class

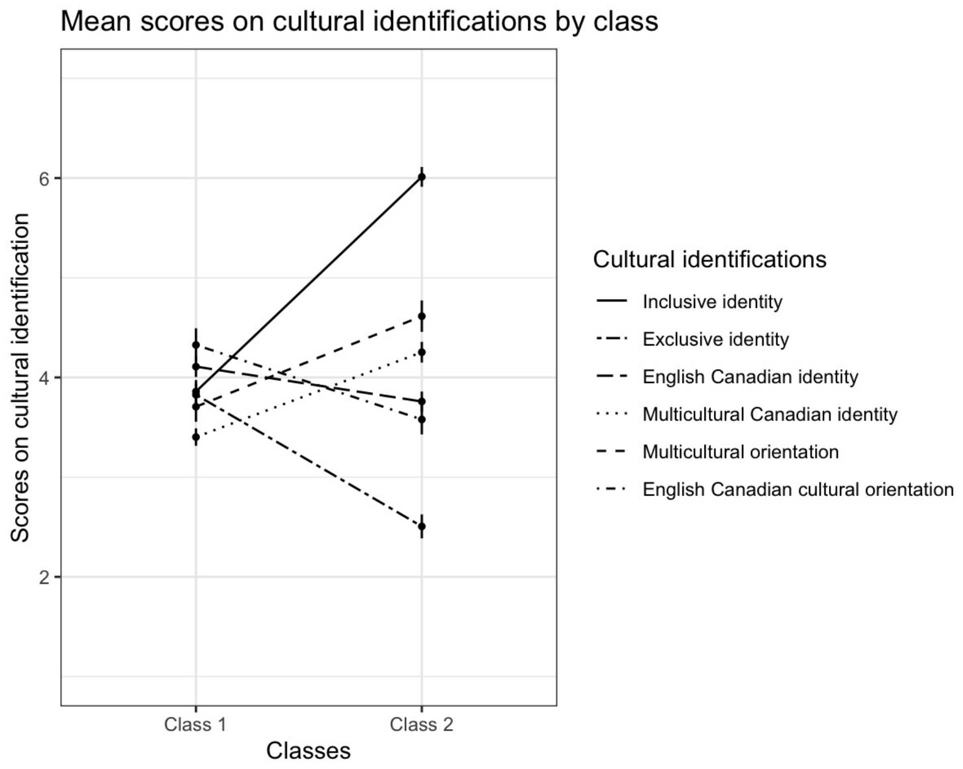

3. **Subsample of participants with parents whose language (for both parents) was English (N=172)**

```
Estimated class population shares
 0.62; 0.38

=========================================================
Regression coefficients:
=========================================================
2 / 1
              Coefficient  Std. error  t value  Pr(>|t|)
(Intercept)    -2.08531     1.70655   -1.222     0.225
sexFemale      -0.29077     0.59271   -0.491     0.625
age            -0.04927     0.02524   -1.952     0.054
cfs             0.70284     0.33184    2.118     0.037
=========================================================

number of observations: 172
number of estimated parameters: 70
residual degrees of freedom: 102
maximum log-likelihood: -1591.466

AIC(2): 3322.931
BIC(2): 3543.256
X^2(2): 304004 (Chi-square goodness of fit)

Mean posterior probabilities:
Class 1   0.94
Class 2   0.93
Across all classes 0.93

Relative entropy: 0.77
```

Visualization of response patterns by class

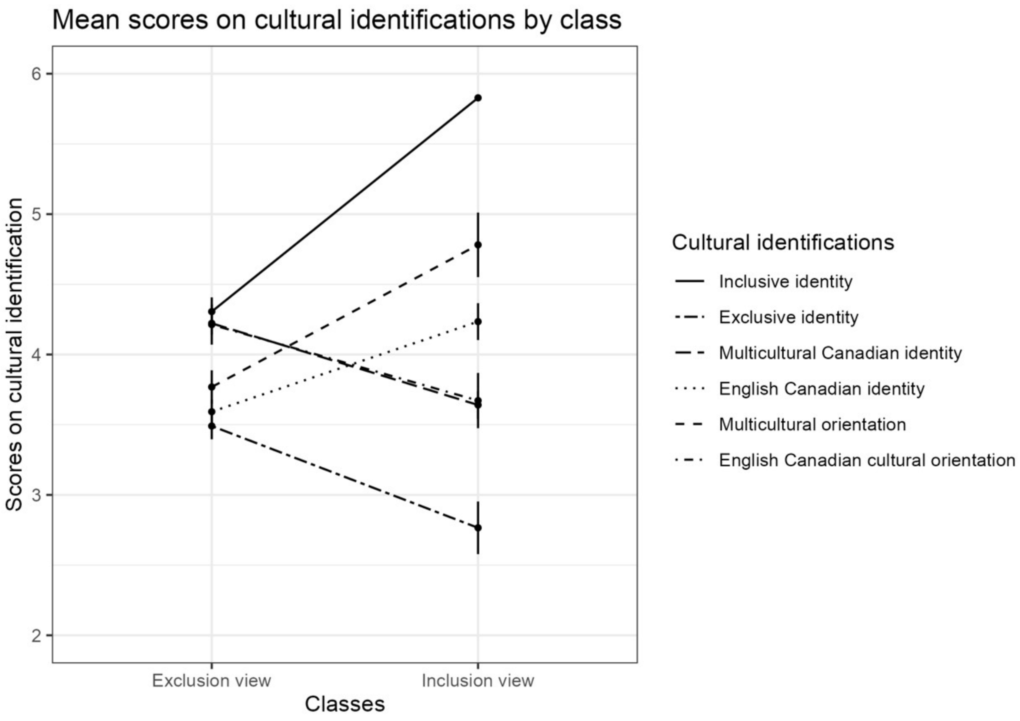

II. CFA for Inclusion Exclusion subscales

Questions
In your opinion, to what extent are the groups below representative of the Canadian identity?
Inclusion
All of Canada's melting pot of citizens (inexis_1)
The various ethnic groups that live in Canada (inexis_2)
The diverse cultural groups that compose the country (inexis_4)
The various linguistic groups that live in the Canada (inexis_7)
All of Canada's diverse population (inexis_7)
Exclusion
Only groups of people who are of European descent (intexis_3)
Groups of people who share similar cultural characteristics with the first settlers in Canada (inexis_5)
Only those who are native speakers of English (inexis_6)
Only people who were born in the Canada (inexis_8)
Group members who embrace core Canadian values (such as equality, freedom, etc.) (inexis_10)

Latent Variables:

1. Inclusion:
    - All the item loadings for the Inclusion latent variable (inexis_1, inexis_2, inexis_4, inexis_7, inexis_9) are statistically significant with very low p-values ($p < 0.001$). This indicates that these items strongly contribute to the Inclusion construct.
    - The standardized loadings (Std.all) are relatively high for all items, with values ranging from 0.631 to 0.903, suggesting good item-to-construct associations. The highest loading is for inexis_9 (0.903), and the lowest is for inexis_1 (0.631). This shows that inexis_1 is less strongly associated with the Inclusion construct, but still contributes significantly.
2. Exclusion:
    - For the Exclusion latent variable, the item inexis_10 has a very small loading of 0.014 (Std.all), which is nearly zero and non-significant ($p = 0.859$). This suggests that inexis_10 does not meaningfully contribute to the Exclusion latent variable and may need to be reconsidered in the model.
    - Other items (inexis_3, inexis_5, inexis_6, and inexis_8) have relatively high and significant loadings, ranging from 0.533 to 0.877 (Std.all), indicating that they strongly contribute to the Exclusion construct. The highest loading is for inexis_6 (0.877).

Covariances:

- There is a significant negative covariance between Inclusion and Exclusion (Estimate = -1.043, $p < 0.001$). This suggests that higher levels of inclusion are associated with lower levels of exclusion, indicating that these two constructs might be inversely related in the

model. The negative covariance is reflected in both the latent variable level (-0.590 standardized) and the raw score level.

Variances:

- The variances of the residuals for the items are significant, with all p-values being less than 0.001, indicating that the residuals are not due to random error.
  - For Inclusion, the variance of the residual for inexis_1 (1.978) is the highest, suggesting that it has relatively high unexplained variance compared to other items in the Inclusion subscale.
  - For Exclusion, the residual variance of inexis_10 is particularly high (2.246), which could indicate that this item is either poorly measured or not well explained by the Exclusion construct.

Overall Model Fit:

- Given the significant estimates, standardized loadings, and variances, the model seems to fit well, suggesting that the two latent variables (Inclusion and Exclusion) are well-defined. However, the non-significant loading for inexis_10 should be addressed in further model revisions or sensitivity analyses.

The CFA model supports the hypothesis that both Inclusion and Exclusion are valid latent constructs, but the item inexis_10 does not seem to measure the Exclusion construct effectively. Further refinement of the model, such as removing or re-assessing inexis_10, might improve the model fit. Additionally, the negative covariance between Inclusion and Exclusion supports the idea that these two constructs are inversely related.

To compare models fit, we ran CFA without problematic item.

The model presented without the item inexis_10 shows a similar pattern to the previous model, but with notable differences in fit and parameter estimates.

Model Fit Comparison:

1. Chi-Square Test:
   - The user model (without inexis_10) has a Chi-square statistic of 44.229 ($p = 0.014$), indicating a significant model fit. However, the baseline model shows a much higher Chi-square of 1198.425 ($p < 0.001$), which implies that the user model (without inexis_10) fits the data significantly better than the baseline model.
   - The scaling correction factor for the user model is 1.010, which is lower than the baseline model's 1.409, suggesting a better adjustment for non-normality in the user model.
2. CFI and TLI:
   - The Comparative Fit Index (CFI) for the user model is 0.984, and the Tucker-Lewis Index (TLI) is 0.978, both of which are above the commonly accepted

    thresholds for good fit (CFI > 0.95, TLI > 0.95). This indicates that the model, even without inexis_10, provides a good fit to the data.
    - In comparison, the baseline model has slightly lower indices (CFI = 0.978, TLI = 0.970), suggesting that removing the problematic item inexis_10 leads to a better-fitting model.
3. RMSEA and SRMR:
    - The Root Mean Square Error of Approximation (RMSEA) for the user model is 0.060 (90% CI: 0.026 - 0.090), which is within the acceptable range (RMSEA < 0.08), suggesting good fit. The SRMR value is 0.044, which is also well below the threshold of 0.08, indicating good model fit.
    - The baseline model has an RMSEA of 0.060 and a slightly higher upper bound for the confidence interval (0.090), which also suggests a reasonable fit but still worse than the user model.
4. Akaike Information Criterion (AIC) and Bayesian Information Criterion (BIC):
    - AIC and BIC values for the user model (5688.560 and 5750.551) are similar to the baseline model, but since the user model fits better as indicated by the fit indices, this suggests a more parsimonious model.

Parameter Estimates:

- The factor loadings for the inclusion and exclusion subscales remain high and statistically significant.
    - For the Inclusion latent variable, the loadings for inexis_1, inexis_2, inexis_4, inexis_7, and inexis_9 are all significant ($p < 0.001$) and have standardized loadings between 0.631 (for inexis_1) and 0.903 (for inexis_9), which suggests a strong contribution of these items to the Inclusion factor.
    - Similarly, the Exclusion latent variable has strong loadings, with inexis_3, inexis_5, inexis_6, and inexis_8 all showing significant loadings between 0.533 and 0.877 (Std.all), suggesting that these items are good indicators of the Exclusion factor.
- The negative covariance between Inclusion and Exclusion remains significant (Estimate = -1.043, $p < 0.001$), indicating that the two latent constructs are inversely related, which is consistent with the theoretical expectation.

Residual Variances:

- The residual variances are statistically significant for all items, indicating that the items do not perfectly load onto their respective latent variables, which is typical for real-world data. The residual variances for Inclusion items range from 0.136 (for inexis_4) to 0.601 (for inexis_1), suggesting varying levels of unexplained variance. Similarly, the residual variances for Exclusion items range from 0.231 (for inexis_6) to 1.000 (for inexis_10), showing that some items (especially inexis_10) are not well explained by the model.

Conclusion:

The model excluding inexis_10 appears to fit the data better than the baseline model and provides a good fit based on the fit indices (CFI, TLI, RMSEA, SRMR). The factor loadings for the inclusion and exclusion subscales remain strong and consistent, reinforcing the validity of these constructs.

The negative covariance between Inclusion and Exclusion supports the theoretical relationship between these two constructs, suggesting they are inversely related.

Given that the inexis_10 item had a very small and non-significant loading in the original model, its removal improves the overall model fit, suggesting that this item may not be a valid indicator for the Exclusion latent variable.

The exlusion subscale without inexis_10 could be better suited for further analyses. Removing this item enhances model fit and produces more meaningful results. Further validation of the remaining items could help refine the model even further.
